# Adaptation and Bonding of Bulk-Fill Composites in Deep Preparations

**DOI:** 10.3390/ma18163790

**Published:** 2025-08-12

**Authors:** Juman Al-Haddad, Nafiseh Najmafshar, Andre V. Ritter, Alireza Sadr

**Affiliations:** Biomimetics Biomaterials Biophotonics Biomechanics & Technology (B4T), Department of Restorative Dentistry, University of Washington, 1959 NE Pacific St., Seattle, WA 98195, USA; jhaddad@uw.edu (J.A.-H.); nafiseh@uw.edu (N.N.); avritter@uw.edu (A.V.R.)

**Keywords:** dentin bonding, bulk-filled composite, dual-cure, micro-tensile bond strength, optical coherence tomography, self-adhesive resins

## Abstract

Polymerization shrinkage in resin-based composites can lead to gap formation at the tooth–restoration interface, potentially compromising the long-term success of restorations. Bulk-fill composites have been developed to reduce shrinkage stress, but their adaptation and bond strength—especially in deep cavities—remain areas of concern. This study investigated the adaptation and bond strength of a newly developed dual-cure bulk-fill composite in 4 mm deep preparations compared to light-cured and self-adhesive bulk-fill composites in six groups. Standard composite molds were used to observe and measure sealed floor area (SFA%) of the composite after the polymerization process under optical coherence tomography (OCT) imaging. Micro-tensile bond strength (MTBS) testing was conducted in extracted human teeth. OCT showed that the prototype dual-cure composites had the lowest gap formation during polymerization (SFA 91%), while the self-adhesive composite demonstrated the highest debonding from the cavity floor (SFA 26%, *p* < 0.001). For MTBS analysis, the lowest mean bond strength was recorded for the self-adhesive composite (~21 MPa) and the highest for a light-cured bulk-fill (~50 MPa, *p* < 0.05). Overall, the dual-cure bulk-fill composites exhibited less gap formation than the light-cured ones. The prototype dual-cure material with 90 s waiting before light-curing showed the best adaptation. However, these differences were not reflected in the bond strength values to the cavity floor dentin using the universal adhesive used in the current study, as the light-cured composite showed the highest bond strength values. The self-adhesive composite showed the poorest results in both experiments, indicating that the application of a bonding system is still necessary for better adaptation and bonding to the cavity floor dentin.

## 1. Introduction

Resin-based composites are increasingly used for direct posterior restorations due to their aesthetic appearance, ease of use and patients’ preferences [1]. Composites are made from the blending of silanized fillers and a resin matrix, polymerized by a free-radical reaction, and activated by curing light. Current resin-based composites undergo shrinkage during setting or polymerization, which may have undesired clinical consequences [2]. During polymerization, the material shrinks, causing a reduction in the composite volume which in turn leads to gap formation. The gaps formed are undesirable and can lead to clinical problems [1,3,4,5,6].

**Figure 1:**
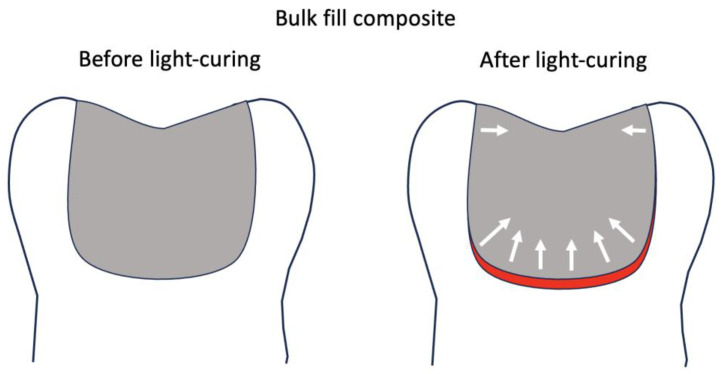
A high C-factor situation is shown in a bonded occlusal preparation. The illustration on the left shows the restorative material fully adapted to the cavity preparation prior to light-curing. The illustration on the right shows gap formation, shown in red, primarily along the deep cavity floor of the cavity post light-curing. The arrows represent shrinkage stress vectors intensity and direction.

Historically, incremental placement of composite has been recommended to control the stress developed during contraction. Bulk-fill composites were introduced claiming to have properties that overcome the polymerization shrinkage. Even though bulk-fill composites have shown greater adhesion and bond strength than conventional flowable composites when placed as bulk in 4 mm deep preparations, there were still interfacial gaps that caused a compromise in the sealing of the composite to the cavity surface, regardless of the composite type [7,8]. Research has shown that despite improving the composition of new bulk-fill composites, gaps tend to form mostly at the line-angles or at the cavity base, ranging from less than a micrometer up to several tens of micrometers in size [9,10,11]. A key contributing factor to this phenomenon is the configuration factor, or “C-factor,” which refers to the ratio of bonded to unbonded surfaces in a tooth cavity. High C-factor cavities—such as deep occlusal preparations—limit the ability of the composite resin to relieve internal stress during polymerization shrinkage, thereby increasing the likelihood of gap formation. The shrinkage stress vectors are the largest at the deepest area of the preparation in a light-cured composite, which results in debonding from deep dentin (Figure 1) [12,13].

Clinically, these micro-gaps are undetectable by the conventional diagnostic methods, such as radiographic films, which suffer from image super imposition and lack of accuracy on the micron-scale. Conventionally, dye-penetration leakage tests and microscopic assessment of the interface have been employed for in vitro detection of such defects. Those destructive methods require sectioning of the teeth to evaluate the interface and cannot specify the origin and timing of gap formation. In this regard, incorporation of newer diagnostic technologies for research on adaptation of restoration, such as 3D imaging by X-ray micro computed tomography (micro-CT) and optical coherence tomography (OCT) have gained increasing attention in adhesive dentistry research [3,5,6].

Swept-source optical coherence tomography (SS-OCT) uses non-ionizing radiation for non-destructive testing of dental restorative materials. It has shown the capability to detect the gap along the cavity floor of composite restorations [3,14]. It has become a promising imaging modality in dentistry, one that does not require the cutting and processing of the specimens as it allows the visualization of microstructures of tissues and biomaterials in real time [15]. This technology demonstrated a remarkable capability with high sensitivity and accuracy in detection and quantification of gaps as small as a few micrometers at the bottom of composite restorations; SS-OCT is a modern research tool in the dental research field, used to evaluate and visualize caries, fractures and interfacial gaps in restorations [5,6]. The use of an OCT in a clinical setting may be implemented in assessing different cavity preparations, composite filling techniques and restorative materials [10].

Currently, there is concern regarding the formation of gaps between a bulk-fill composite and the deep dentin due to polymerization shrinkage, poor copolymerization and lack of bond strength [16]. Long-term clinical research data and evidence on the effectiveness of bulk-fill materials do not exist. However, it is known that restorative material defects and gaps eventually lead to secondary caries, restoration failure, fracture of a tooth, or increased sensitivity in a vital tooth. Despite various efforts to improve bulk-fill composite performance, there remains a lack of consensus on how different formulations—especially dual-cure flowable materials—affect gap formation and bond strength in high C-factor cavities. The difference between dual-cure bulk-fill composites and traditional composites is of interest. A comparative study examined the qualitative and quantitative differences between two dual-cure composites and other light-cure composites [12,13]. The results indicated that dual-cure composites, depending on their chemical initiator system, exhibited moderately paced polymerization kinetics compared to the light-cure composites at the same depth. In another study, light-cure bulk-fill composites showed variations in the speed of polymerization based on depths, concluding that polymerization kinetics affect contraction stress and clinical performance [17].

Flowable composites could be used in restoration of non-carious cervical lesions and minimally invasive occlusal cavity preparations [18]. There seems to be an advantage with the flowable bulk-fill approach in terms of internal adaptation. Most of the current flowable bulk-fill composites require an additional “capping” layer of paste-type hybrid composite for stress-bearing occlusal surfaces A prototype experimental formulation of the dual-cure flowable using 80 nm spherical zirconia fillers has been developed with claims to improve polish-ability and wear resistance, reducing the need for a capping hybrid composite. One advantage of a flowable composite for the bulk-filling technique is thought to be in its pre-gel viscoelastic behavior, allowing relaxation within the resin matrix and reducing the chance of volumetric shrinkage turning into harmful stresses [12,13]. However, the filler composition affects the polymerization properties of the composites. Therefore, the polymerization behavior and gap formation of the new composite need to be investigated.

In addition to marginal adaptation, the bond strength of the bulk-fill composites to the deep dentin has been a point of concern. The micro-tensile bond strength (MTBS) test has been reportedly used in many studies to evaluate the bond strength and the effectiveness of bonding systems between the cavity preparation and the restorative material used. The MTBS test was used in many studies to test the adhesion strength between different composite resin materials and dentin by evaluating the amount of force it requires for restorations to de-bond from the cavity floor [19,20]. In previous studies, the bond strength of a bulk-fill composite was enhanced when the same composite was placed incrementally, indicating the detrimental effect of shrinkage stress on the bond strength [5].

Over the past few years, manufacturers have attempted to develop self-adhesive bulk-fill composites. Such materials seem to be ideal in terms of saving clinical chair time, but the literature consistently reports poor performance when compared to separate adhesive and composite combinations. In a comparative study between a conventional bulk-fill composite and a self-adhesive bulk-fill composite, results showed that using a bonding system is favorable in clinical conditions to avoid gap formation between the cavity prep and the composite material [21]. To date, few studies have combined both OCT-based gap evaluation and bond strength testing to comprehensively assess the internal adaptation of dual-cure bulk-fill composites. This study addresses that gap by examining both outcomes in high C-factor cavity conditions.

Therefore, this study investigated the internal adaptation and micro-tensile bond strength of bulk-fill composites to deep structure in high C-factor cavities. The null hypotheses were that (1) there would be no differences in internal adaptation between the tested bulk-fill composites, and (2) there would be no differences in MTBS values between the tested bulk-fill composites.

## 2. Materials and Methods

### 2.1. Experimental Groups

Six bulk-fill composite products were tested: Bulk EZ Plus prototype (BEZP, Zest Dental Solutions, Carlsbad, CA, USA), Surefil SDR Flow+ (SDRF, Dentsply, Milford, DE, USA), Surefil One self-adhesive capsule (SONE, Dentsply Sirona, Milford, DE, USA), Filtek One (FTON, 3M, St. Paul, MN, USA), Hyperfil (HYPF, Parkell, Edgewood, NY, USA), and Sonicfill 3 (SNCF, Kerr, Brea, CA, USA). Material composition and special instructions are found in Table 1.

Composites were inserted into the cavity molds and prepped human teeth in bulk following a standard bonding protocol where necessary. An LED light-curing unit (Paradigm Deep Cure, 3M, St. Paul, MN, USA) at 1470 mW/cm^2^ was used for light polymerization. For composites which required a bonding system, Scotchbond Universal (3M, St. Paul, MN, USA) universal one-step bonding system was used for all of the specimens in both OCT and MTBS experiments. It is important to note that the 90 s auto-polymerization waiting time before light-curing was applied only to the BEZP prototype, as per the manufacturer’s recommendation, while HYPF was light-cured, with no time allotted for self-cure.

### 2.2. Molds, Teeth and Cavity Preparations

Standard composite molds 4 mm in depth were used to observe and record the Sealed Floor Area percentage (SFA%) of the bonded composite during the polymerization process using OCT (Octina prototype, Yoshida Dental, Tokyo, Japan). Flowable composite (Estelite Universal Flow, Tokuyama Dental, Tokyo, Japan), containing 71% by weight (53% by volume) silica-zirconia and silica-titania filler, was used to create a cavity mold using 3 × 3 × 4 mm dimensions (W × L × D), as described previously [1,22].

For the MTBS test, human teeth were collected, properly cleaned, and mounted. This study was conducted according to the ethical guidelines set by the University of Washington Institutional Review Board, which waived the approval requirement for the use of deidentified human teeth extracted as part of routine dental treatment. The teeth were trimmed down occlusally to create a uniform surface while maintaining enamel integrity. The occlusal surfaces were then covered with 1 mm of Clearfil Photo Core composite [4.4 g/2 mL] (Kuraray Noritake Dental, Tokyo, Japan). The 1 mm layer of composite was added to ensure appropriate depth of prep while avoiding exposure to the pulp. A prep of 3 × 3 × 4 mm in dimensions was then created in each tooth.

### 2.3. Optical Coherence Tomography

**Figure 2:**
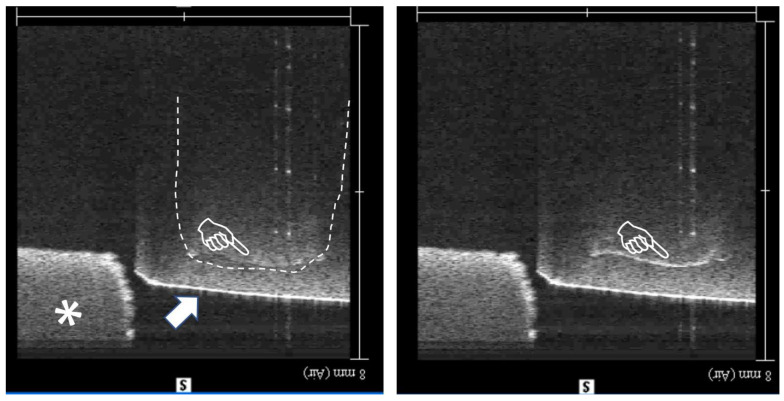
OCT 2D cross-sections for measurement of debonding from cavity floor for each specimen. Three-dimensional images were evaluated both prior to polymerization and afterwards; dotted line: composite restoration margins; Arrow: outside surface of the bottom of the specimen; finger pointer: debonded cavity floor, measuring SFA 100% at this cross-section; asterisk: specimen holder.

OCT has been previously used to analyze gap formation on standard composite molds with cylindrical cavities (3 mm D × 4 mm H), which mimic a cavity preparation [12,13]. In this study, the cavity molds were prepared in 3 × 3 × 4 mm dimensions. Figure 2 shows OCT images of one the specimens used in this study both pre and post polymerization. A thick white line after polymerization indicates full debonding from the cavity floor. The internal adaptation was evaluated using an image analysis software (ImageJ version 1.53t, National Institutes of Health, Bethesda, MD, USA) according to the method described in previous studies, based on the sealed floor area percentage (SFA%) values which were calculated considering the area proportion of the debonding (gap) over total cavity floor area, as described previously [3,12]. Video S1 shows the real-time capture of debonding during photo polymerization of the specimen shown in Figure 2.

### 2.4. Micro-Tensile Bond Strength Test

Human posterior teeth were collected from clinics in the Seattle metropolitan area after extraction for periodontal, orthodontic, or surgical reasons. Before proceeding with the study, teeth were examined to ensure they were intact, non-restored and non-carious. From the time of extraction, teeth were stored in water at 4 °C for less than two months. The teeth were mounted in epoxy resin (Total Boat, Bristol, RI, USA) and the cusps were flattened using a low-speed diamond saw (Buehler, Lake Bluff, IL, USA) under abundant cooling water to form a flat occlusal surface. To ensure that a 4 mm deep preparation is reached without compromising the dentin leaving a very thin layer or causing exposure of the pulp chamber, a 1 mm thick layer of hybrid resin composite was placed on the occlusal flat surface, using one-step bonding agent, and cured for 20 s.

An occlusal class one cavity (3 × 3 × 4 mm^3^) was prepared in each tooth using a long flat-end cylindrical diamond bur (Shofu, Kyoto, Japan) under cooling water to obtain a flat cavity floor. The cavities were treated using standard bonding protocol as in the gap experiment according to the manufacturer’s instructions and restored in bulk to obtain six experimental groups.

MTBS beams were prepared as follows: after 24 h of storage in 37 °C water, teeth were sectioned using a low-speed diamond saw under abundant cooling water to create beams approximately 0.7 mm in thickness. Those beams were later cut using green banded diamond burs under slow speed and water into an hourglass shape, leaving 0.7 mm in the middle where stress was concentrated. The marginal beams were excluded from this study. The specimen thickness and the width of bonded area after trimming were verified and recorded using a micrometer caliper (Mitutoyo, Tokyo, Japan). Four central beams with no defects were selected for the MTBS test at a crosshead speed of 1 mm/min (Bisco Microtensile Tester, Schaumburg, IL, USA). The beams were then placed on the MTBS machine for testing.

For statistical analysis purposes, each tooth was considered as a statistical unit and therefore, all the values obtained from each tooth were averaged. Pre-test failures where composite has separated from dentin prior to testing were recorded and reported but excluded from statistical analysis. Pre-test failures (PTF) where composite had separated from dentin prior to testing were recorded as 0.0 values. The statistical analysis for MTBS was performed twice, one excluding the PTF and another including the PTF.

Subject to data normality and variance requirements, the results were statistically compared; OCT data was analyzed by non-parametric tests, Kruskal–Wallis (KW) and Mann–Whitney U for pairwise comparisons while MTBS data was analyzed by one-way ANOVA with post hoc Bonferroni comparisons at 0.05 significance level.

## 3. Results

### 3.1. OCT Results

**Figure 3:**
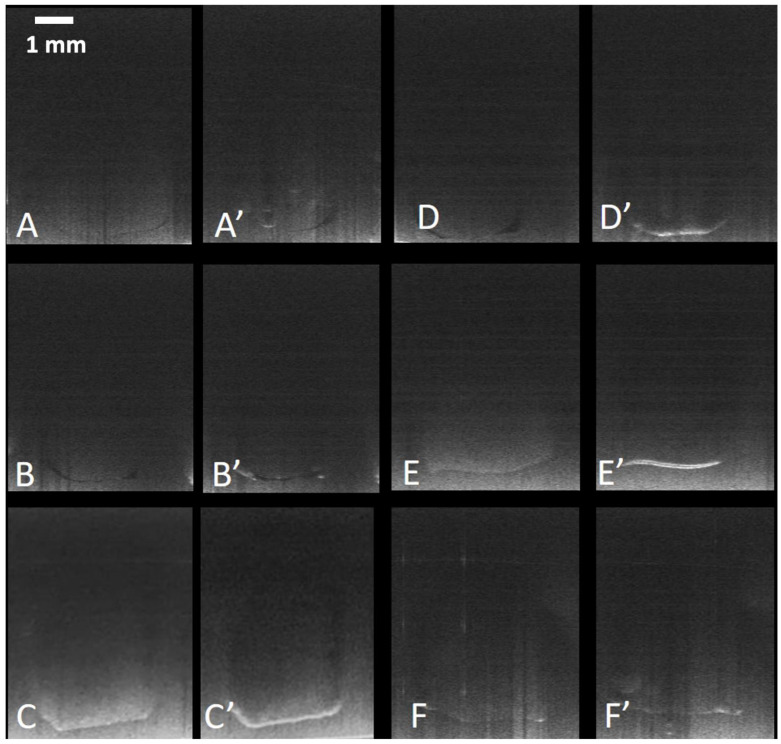
pre-cure (**A**–**F**) and post-cure (**A**’–**F**’) for each experimental product. (**A**,**A**’) = BEZP, (**B**,**B**’) = SDRF, (**C**,**C**’) = SONE, (**D**,**D**’) = FTON, (**E**,**E**’) = HYPF, (**F**,**F**’) = SNCF. Debonding shows as a high intensity (white) line along the cavity floor, while the dark line represents a thick bond layer. Different groups appear differently under OCT due to their optical properties. Horizontal bar scale = 1 mm.

Representative OCT images for each group are presented in Figure 3. SFA% and KW Mean Ranks are summarized in Table 2. The KW test showed that there was a significant difference in gap formation among the groups (*p* = 0.000). BEZP followed by HYPF had the lowest nominal values for gap formation during polymerization; pairwise comparisons showed that BEZP (SFA 91%) significantly different from all other groups (*p* < 0.05). On the other hand, SONE demonstrated the highest debonding and lowest sealing of the cavity floor (SFA 26%) which was also different from all other groups (*p* < 0.05). There was no statistically significant difference (*p* > 0.05) between HYPF (SFA 77%), SDRF (SFA 70%), FTON (SFA 64%) and SNCF (SFA 76%).

### 3.2. MTBS Results

Table 3 shows MTBS test values for each group, excluding PTF values of zero from the data, while Table 4 presents the data including PTF. One-way ANOVA showed a significant difference among the groups (F = 8.774, *p* = 0.000). The lowest mean bond strength was recorded for SONE (21.5 ± 8.8 MPa) and the highest for SDRF (49.8 ± 10.5 MPa), which was significantly different from all the groups. There was no statistically significant difference among other groups (*p* > 0.05). However, when the PTF values were taken into account, the MTBS values were reduced for all the groups while the general trend did not change. The only statically significant difference in this case was detected between SDRF and SONE (F = 3.3049, *p* = 0.009).

## 4. Discussion

Since OCT is nondestructive, it enables detection of gaps developed during light-curing as well as pre-existing defects. To create standard cavities to simulate large C-factor conditions clinically, simulated composite cavities were used in this study, as described previously [6,13].

MTBS is the most performed testing procedure in adhesive dentistry, which gained popularity due to its enhanced accuracy in detecting differences between the bonding ability of materials and its higher discriminative power than traditional tests [23]. The approach of combining objective OCT imaging in simulated cavities and the MTBS test on human teeth sheds light on the performance of each material, with both variable thoughts to contribute to understanding the clinical success of the different dental materials [1].

SNCF employs a specialized handpiece that transmits sonic energy at adjustable intensities to enhance the placement of composite resin. During activation, the sonic energy triggers a built-in modifier that temporarily lowers the composite viscosity aiming to allow it to flow more easily and adapt to cavity walls. Once the sonic activation stops, the material reverts to a higher-viscosity state for sculpting and shaping. According to the manufacturer, this approach is designed for the bulk-filling of large cavities while minimizing the risk of void formation. However, voids were observed at the dentin interface in the microscopic analysis in a previous study which was in line with the current OCT observation with SNCF offering no advantage over other bulk-fill materials.

HYPF employs enhanced protection techniques by self-curing allowing the composite to adapt to the cavity prep floor and walls, significantly lowering gap formation, microleakage, and post-op sensitivity. The composite also exhibits complete polymerization no matter how far it is from the light-cure, allowing for a bulk-fill technique in challenging clinical cases. This was reflected in the results seen in both experiments. HYPF exhibited minimal gap formation, high SFA%, and relatively strong bond strength when tested with the MTBS test.

FTON contains Addition-Fragmentation Monomer (AFM), which is reported to fragment to relieve stress and then re-polymerize in a lower stress state, and Aromatic Urethane DimethylAcrylate (AUDMA), which has bigger molecules that can limit space or shrinkage zones during polymerization, avoiding polymerization shrinkage and making it suitable for bulk-filling. FTON exhibited more gap formation during the OCT experiments, and voids within the composite were present.

SONE, the self-adhesive composite [24], showed more debonding from the cavity preparation in both the OCT and MTBS experiments, compared to composites that used an adhesive system [16]. Even though the self-adhesive approach could be beneficial in terms of chair-side time due to elimination of the bonding step [20], it is recommended that clinicians use a separate bonding system for better long-term results. A previous report demonstrated that SONE showed higher bond strength to flat dentin than that to the dentin at the base of a bulk-filled cavity floor and attributed the lower bond strength to the effects of polymerization shrinkage stress of the hybrid restorative material, further indicated the need for a bonding system to ensure the success of the restorative material [24,25].

There have been attempts to increase the translucency of light-cure bulk-fill composites due to poor light attenuation through the restoration and insufficient dual-cure throughout the restoration [13,17]. In this study, de-bonding was noted in both light-cure and dual-cure composites. However, BEZP exhibited superior behavior due to dual-cure setting time. It is noteworthy that a 90 s wait is required for the chemical-cure reaction to proceed before light-curing for BEZP, while for HYPF, light-curing is indicated as soon as the material is dispensed. Based on the results, it appears that allowing time for an effective chemical curing of the dual-cure material such as in BEZP prior to light irradiation is advantageous over immediate light-curing of the dual-cure composite [4].

It is important to note that in terms of debonding, there was a difference between the OCT and MTBS tendencies. While BEZP showed the best adaptation (lowest gap formation) under OCT, its MTBS values were not the highest. This discrepancy highlights that excellent adaptation does not necessarily translate to high bond strength. The interface quality and chemical compatibility with the adhesive also play a crucial role, as seen in SDRF’s strong MTBS performance despite more gap formation. OCT predominantly showed that BEZP had the highest sealing and lowest gaps, while the MTBS test showed that SDRF had the highest bond strength, indicating better bonding than the other groups. This is in line with previous findings that SDRF was the only light-cured bulk-fill composite material, which did not show a reduction in MTBS between flat dentin and high C-factor cavity floor [22]. This difference was attributed to the copolymerization between the light-cured composite and the light-cured bonding agent, resulting in the formation of better integrity at the composite-adhesive interface. From a clinical perspective, the presence of interfacial gaps can allow for fluid and bacterial ingress, which may result in microleakage, post-operative sensitivity, pulp inflammation, or secondary caries. Although this study did not directly assess leakage or bacterial penetration, the observed gaps—especially in self-adhesive materials like SONE—highlight a potential risk for clinical failure. These findings support the importance of both minimizing polymerization shrinkage and ensuring strong adhesive bonding to reduce gap formation and improve long-term restoration success.

BEZP showed lower bond strength to the cavity floor dentin in comparison with SDRF light-cured composite, using the universal adhesive in the current study. A large number of pretest failures were detected in BEZP, which was not expected given its excellent adaptation noted from the OCT experiment. These failures were due to adhesive failure between the composite and the universal bonding agent. It appears that although the chemical cure mechanism in BEZP reduced the pulling away of the composite from the bonding layer during its polymerization, the quality of copolymerization with the universal bond was not as good as that in comparison with the light-cured composite SDRF. Due to concerns regarding the incompatibilities between simplified adhesives and dual-cure composites [26], we performed additional experiments using a two-step self-etch adhesive bonding system (Clearfil SE Bond 2, Kuraray Noritake, Tokyo, Japan) and BEZP. The two-step bond has a water- and solvent-free separate bonding agent, which is less acidic than the universal blends. The bond strength to human dentin in this case improved and reached 35.8 ± 5.4 MPa with no pretest failures, indicating a possible incompatibility between the universal adhesive and the dual-cure composite in MTBS test. These findings suggest that the lower performance of some materials, such as BEZP, may stem from chemical incompatibility with the adhesive rather than effects of shrinkage stress. In contrast, the poor performance of SONE is likely attributed to its formulation and the absence of a bonding step.

Further research should elucidate the relationship between polymerization shrinkage and compatibility of the composite products with the different bonding systems. This is important because clinicians need to be aware of the limitations of the use of different bonding systems with composites. Further studies need to be conducted to better understand possible adverse interactions between BEZP and various bonding agents. It is suggested that when clinicians choose a dual-cure composite, a compatible bonding system should be selected. A limitation of the current study is the lack of direct measurement of polymerization shrinkage stress. Future studies should incorporate techniques such as Raman spectroscopy to better understand the conversion ratios and stress development mechanisms contributing to gap formation in bulk-fill composites.

## 5. Conclusions

This study was motivated by the need to better understand the relationship between internal adaptation and bond strength in dual-cured and light-cured bulk-fill composites, particularly in deep, high C-factor cavity conditions. The dual-cure bulk-fill composites exhibited better adaptation and less gap formation, with the BEZP that allows the 90 s waiting for chemical curing mechanism prior to irradiation showing the best sealing. The difference was not reflected in the bond strength values with the universal adhesive used in the current study, as the SDRF light-cured bulk-fill showed the highest bond strength to the cavity floor and least-frequent pretest failure. The self-adhesive composite SONE showed the poorest results in both experiments, indicating that application of a bonding system is still necessary for better adaptation and bonding to the cavity floor and further development of self-adhesive composite technology is warranted. In addition, the compatibility between the dual-cure composites and the universal bonding systems remains a concern that can be further explored in future studies. OCT methodology is a unique nondestructive tool for observation of shrinkage stress defects in composite materials, utilized in this study to properly understand cavity-surface defects.

## 6. Clinical Significance

When placing composite in deep preparation, the dual-cure bulk-fill composites showed better adaptation and less gap formation than the light-cured ones. The self-adhesive composite debonded frequently in both experiments and was inferior to the ones that required a separate adhesive. It is also important to consider the type of bonding system used for each composite type. It is recommended to use a compatible (such as two-step) bonding system for the dual-cure bulk-fill composites.

## Figures and Tables

**Table 1:** The six composite groups used for the purpose of this research along with information regarding the composition of each and special instructions.

Product Viscosity, Delivery and Polymerization	Ingredients	Instructions
Bulk EZ Plus Prototype Dual-cured flowable, automix syringe	Ethoxylated bisphenol A dimethacrylate esters, Triethylene glycol dimethacrylate, Bisphenol A gycidyl methacrylate, Urethane dimethacrylate, Fluoride compound, Glass compound	Use a bonding system Dual-cure, wait time of 90 s of self-cure and then cure with the curing light for 20 s
SureFil SDR Flow + Light-cured flowable, syringe	Polymerizable dimethacrylate resins Polymerizable trimethacrylate resins Polymerizable urethane dimethacrylate	Use a bonding system Cure for 20 s using a curing light
SureFil One Self-adhesive dual-cured paste, capsule	Aluminum-phosphorostrontium-sodium-fluoro-sillicate glass, Water, Highly dispersed silicon dioxide, Acrylic acid, Polycarboxylic acid, Ytterbium fluoride, Bifunctional acrylate, Self-cure initiator, 4-tert-butyl-N,N-dimethylaniline, Iron oxide pigments, Barium sulfate pigment, Manganese pigment, Camphorquinone (photoinitiator), Stabilizer	NO use of a bonding system, self-adhesive material Mix for 10 s, do not manipulate after 90 s, apply and cure using a curing light for 20 s.
Filtek One Light-cured paste composite, Unidose	Silane Treated Ceramic, Aromatic Urethane Dimethacrylate, 1,12-Dodecane Dimethycrylate (DDDMA), Diurethane Dimethacrylate (UDMA), Silane Treated Silica, Ytterbium Fluoride (YbF3), Water, Silane Treated Zirconia	Use a bonding system Cure for 20 s
Hyperfill by Parkell Dual-cured flowable, automix syringe	Silane treated glass fillers, initiators, and stabilizers, Uncured Methacrylate Ester Monomers, Benzoyl peroxide	Use a bonding system Cure for a minimum of 40 s using a curing light Dual-cure. You can let the composite self-cure for 20 s but it is not necessary for polymerization
Sonicfill 3 UltraSonic Light-cured paste composite, unidose with dispensing handpiece	Silicon dioxide, Glass, oxide, chemicals, Poly(oxy-1,2-ethanediyl), α,α’-[(1-methylethylidene)di-4,1-phenylene]bis[ω-[(2-methyl-1-oxo-2-propen-1-yl)oxy], Ytterbium trifluoride, 2,2′-ethylenedioxydiethyl dimethacrylate	Use a bonding system Cure for 20 s using a curing light Use an air-driven dispensing handpiece

**Table 2:** OCT sealed floor area percentage results summary. Non-parametric Kruskal–Wallis test with Mann–Whitney U tests were performed for comparison of ranks among groups (Chi square = 27.3, *p* < 0.001). The superscripts (a, b, c, ab) indicate trends observed by each group, in the order presented.

OCT SFA%				
Group	N	Mean	Std. Deviation	Kruskal–Wallis Mean Ranks
BEZP	10	91	9	46 ^a^
SDRF	10	70	24	31 ^b^
SONE	10	26	25	9.9 ^c^
FTON	10	64	19	25.2 ^b^
HYPF	10	77	33	39 ^ab^
SNCF	10	76	10	31.9 ^b^

**Table 3:** Mean and standard deviation values from the MTBS test for each group with 8 specimens tested per group. Groups indicated by different superscript letters (a, b) present statistically significant differences (one-way ANOVA with Bonferroni correction, F = 8.774, *p* = 0.000).

MTBS (Excluding PTF)					
Group	N	Mean	Std. Deviation	95% Confidence Interval for Mean	
				Lower Bound	Upper Bound
BEZP ^a^	8	33.7	5.3	29.3	38.1
SDRF ^b^	8	49.8	10.5	41.0	58.6
SONE ^a^	8	21.5	8.8	14.2	28.9
FTON ^a^	8	31.2	12.7	20.6	41.8
HYPF ^a^	8	30.7	7.6	24.3	37.0
SNCF ^a^	8	29.9	6.3	24.6	35.2

**Table 4:** Mean and standard deviation values from the MTBS test for each group, including 0 values for pretest failures. SDRF and SONE indicated by different superscript letters (a, b, ab) were significantly different (one-way ANOVA with Bonferroni correction, F = 3.3049, *p* = 0.009).

MTBS (Including PTF)					
Group	N	Mean	Std. Deviation	95% Confidence Interval for Mean	
				Lower Bound	Upper Bound
BEZP ^ab^	8	19.6	15.2	11.8	27.5
SDRF ^a^	8	31.8	20.5	21.6	42.0
SONE ^b^	8	10.4	11.7	4.8	16.0
FTON ^ab^	8	16.9	16.2	7.6	26.2
HYPF ^ab^	8	16.9	13.7	7.9	25.9
SNCF ^ab^	8	19.8	13.7	12.2	27.4

## Data Availability

The original contributions presented in this study are included in the article/Supplementary Materials. Further inquiries can be directed to the corresponding author.

## Supplementary Materials

The following supporting information can be downloaded at: https://www.mdpi.com/article/10.3390/ma18163790/s1, Video S1: Real-time OCT video of debonding in SONE group.
